# Complete Genome Sequences of Two USA300-Related Community-Associated Methicillin-Resistant Staphylococcus aureus Clinical Isolates

**DOI:** 10.1128/MRA.00404-19

**Published:** 2019-05-02

**Authors:** Jo-Ann McClure, Kunyan Zhang

**Affiliations:** a Centre for Antimicrobial Resistance, Alberta Health Services/Alberta Public Laboratories/University of Calgary, Calgary, Alberta, Canada; b Department of Pathology and Laboratory Medicine, University of Calgary, Calgary, Alberta, Canada; c Department of Microbiology, Immunology, and Infectious Diseases, University of Calgary, Calgary, Alberta, Canada; d Department of Medicine, University of Calgary, Calgary, Alberta, Canada; e The Calvin, Phoebe, and Joan Snyder Institute for Chronic Diseases, University of Calgary, Calgary, Alberta, Canada; Georgia Institute of Technology

## Abstract

USA300 is a predominant community-associated methicillin-resistant Staphylococcus aureus strain causing significant morbidity and mortality in North America. We present the full annotated genome sequences of two methicillin-resistant Staphylococcus aureus isolates related to the USA300 pulsotype with the goal of studying the evolutionary relationships of this highly successful strain type.

## ANNOUNCEMENT

Community-associated methicillin-resistant Staphylococcus aureus (CA-MRSA) pulsed-field gel electrophoresis (PFGE) strain type USA300 has rapidly become the predominant MRSA strain in North America (1–7). The group comprises a family of strains with related PFGE banding patterns belonging to clonal complex 8 (CC8) and multilocus sequence type 8 (ST8) and carrying staphylococcal cassette chromosome *mec* type IV. Although it is the leading cause of MRSA skin and soft tissue infections in North America, USA300 can also cause life-threatening diseases, such as sepsis and necrotizing pneumonia, and is appearing in outbreaks worldwide. Because of the unique success of USA300, there is significant interest in investigating the genetic diversity of this group, as well as in understanding its evolutionary history. We selected two MRSA isolates of human origin that are very closely related to USA300 based on PFGE and molecular analyses, with both being ST8, *spa* type t008, and staphylococcal cassette chromosome *mec* (SCC*mec*) type IV, for whole-genome sequencing. Strain C8879 was collected in 2002, prior to a USA300 outbreak in the Calgary Health Region in 2004 (8), while strain JK3137 was collected in 2011, 7 years after the outbreak. Both strains were from hospital patients. These sequences, spanning a 9-year window, will help increase our understanding of the evolution of this successful lineage.

Overnight bacterial cultures were started from single colonies and grown at 37°C, and then genomic DNA was obtained from the MRSA isolates using a phenol-chloroform extraction. Library preparation, DNA sequencing, contig assembly, and genome circularization were performed at the Génome Québec Innovation Centre in Montreal, Quebec, Canada. Sheared large-insert libraries were generated with Covaris g-TUBEs and the SMRTbell template prep kit version 1.0 and then sequenced with Pacific Biosciences (PacBio) RSII sequencing technology using one single-molecule real-time (SMRT) cell. Contig assembly was done using the RS Hierarchical Genome Assembly Process (HGAP) protocol version 2.3.0.140936.p5 using default settings (9–11). The read quality was ensured by aligning shorter reads on longer reads using Basic Local Alignment with Successive Refinement (BLASR) (9). The finalized genome was generated by circularizing with Circlator version 1.4.1 (12) and adjusted to the origin of replication. Gene annotation was done using the NCBI’s Prokaryotic Genome Annotation Pipeline version 4.1 (13).

Three contigs resulted from the assembly of the MRSA strain C8879 reads. There were 91,261 raw reads covering 1,077,017,883 sequenced bases with an average read length of 11,801 bp, an estimated genome coverage of 348×, and a GC content of 32.81%. The resulting chromosome was 2,832,897 bp long with 2,972 genes identified, of which 2,890 were coding DNA sequences (CDS), 82 were RNA genes, and 74 were pseudogenes. Four contigs were produced from the assembly of the JK3137 reads. There were 105,404 raw reads covering 1,138,170,110 sequenced bases with an average read length of 10,798 bp, an estimated genome coverage of 366×, and a GC content of 32.82%. The resulting chromosome was 2,955,000 bp long with 3,150 genes identified, of which 3,068 were CDS, 82 were RNA genes, and 106 were pseudogenes.

A complete analysis is under way looking at the chromosomal components in each of these strains and how they compare to those in the highly successful USA300 outbreak isolate C2406 (14).

### Data availability.

The chromosomal genome sequences have been deposited at GenBank under the accession numbers CP020956 (C8879) and CP020960 (JK3137) and SRA accession numbers SRX5621936 (C8879) and SRX5627280 (JK3137).
